# Segmented inner plexiform layer thickness as a potential biomarker to evaluate open-angle glaucoma: Dendritic degeneration of retinal ganglion cell

**DOI:** 10.1371/journal.pone.0182404

**Published:** 2017-08-03

**Authors:** Eun Kyoung Kim, Hae-Young Lopilly Park, Chan Kee Park

**Affiliations:** 1 Department of Ophthalmology and Visual Science, College of Medicine, The Catholic University of Korea, Seoul, South Korea; 2 Seoul St. Mary’s Hospital, Seoul, South Korea; Bascom Palmer Eye Institute, UNITED STATES

## Abstract

**Purpose:**

To evaluate the changes of retinal nerve fiber layer (RNFL), ganglion cell layer (GCL), inner plexiform layer (IPL), and ganglion cell-inner plexiform layer (GCIPL) thicknesses and compare structure-function relationships of 4 retinal layers using spectral-domain optical coherence tomography (SD-OCT) in macular region of glaucoma patients.

**Methods:**

In cross-sectional study, a total of 85 eyes with pre-perimetric to advanced glaucoma and 26 normal controls were enrolled. The glaucomatous eyes were subdivided into three groups according to the severity of visual field defect: a preperimetric glaucoma group, an early glaucoma group, and a moderate to advanced glaucoma group. RNFL, GCL, IPL, and GCIPL thicknesses were measured at the level of the macula by the Spectralis (Heidelberg Engineering, Heidelberg, Germany) SD-OCT with automated segmentation software. For functional evaluation, corresponding mean sensitivity (MS) values were measured using 24–2 standard automated perimetry (SAP).

**Results:**

RNFL, GCL, IPL, and GCIPL thicknesses were significantly different among 4 groups (P < .001). Macular structure losses were positively correlated with the MS values of the 24–2 SAP for RNFL, GCL, IPL, and GCIPL (R = 0.553, 0.636, 0.648 and 0.646, respectively, *P* < .001). In regression analysis, IPL and GCIPL thicknesses showed stronger association with the corresponding MS values of 24–2 SAP compared with RNFL and GCL thicknesses (R^2^ = 0.420, P < .001 for IPL; R^2^ = 0.417, P< .001 for GCIPL thickness).

**Conclusions:**

Segmented IPL thickness was significantly associated with the degree of glaucoma. Segmental analysis of the inner retinal layer including the IPL in macular region may provide valuable information for evaluating glaucoma.

## Introduction

Loss of sight exacts a huge economic cost on both the individual and society. Glaucoma is a progressive optic neuropathy associated with damage to the retinal ganglion cells (RGCs). A comprehensive understanding of the timing and pattern of cellular changes leading to death of RGCs might offer new insights into the onset and progression of the glaucoma and ultimately help to develop new therapies to prevent loss of vision in this disease.

Anatomically, the RGC is located across three layers in the inner retina. The retinal nerve fiber layer (RNFL) is formed by the expansion of the RCG axon, the ganglion cell layer (GCL) consists of RGC bodies, and the inner plexiform layer (IPL) is made up of fibers formed by RGC dendrites, axons of bipolar cells, and processes of amacrine cells. In the course of visual transmission, RGC dendrites receive synaptic inputs from bipolar and amacrine cells in the IPL, which are integrated via axons in the optic nerve [1–6]. Numerous histopathological studies have shed light on the changes in inner retinal layers when axonal injury occurs in glaucoma. Agostinone et al reported that glaucoma-induced damage to the RGC causes changes in the axon and soma as well as dendrite shrinkage and synaptic loss, and that dendritic changes precede changes in the other two cellular compartments [7]. Furthermore, there have been reports that axonal injury at the level of the lamina cribrosa blocks axoplasmic transport from the RGC soma to the axon terminal, resulting eventually in death of the RGC [8,9].

A high proportion of RGCs are present at the level of the macula [10]. Advances in imaging equipment have enabled researchers to undertake a more detailed analysis of the inner retina in the area of the macula. Many studies have measured the RNFL and the ganglion cell-inner plexiform layer (GCIPL) thickness in the macula of patients with glaucoma by using the intraretinal segmentation software of Cirrus spectral-domain optical coherence tomography (SD-OCT) [11,12]. However, the Cirrus (Carl Zeiss Meditec, Dublin, CA) SD-OCT software was inadequate for observing dendritic changes in RGCs because the software could not measure the thickness of the GCL and IPL separately. With the recent advances in segmentation software, Spectralis (Heidelberg Engineering, Heidelberg, Germany), SD-OCT enables measurement of each of the retinal layers independently at the macular level, i.e., the RNFL, the GCL, and the IPL, which contain the ganglion cell axons, cell bodies, and dendrites, respectively [13].

The present study examined the changes of RNFL, GCL, IPL, and GCIPL thicknesses in glaucoma and compared the structure-function relationships between the thicknesses of three layers and visual field (VF) sensitivities by measuring segmented RNFL, GCL, IPL, and GCIPL thicknesses using SD-OCT in macular region.

## Methods

### Study population

This observational cross-sectional study enrolled 85 patients with preperimetric to advanced open angle glaucoma seen by a glaucoma specialist (CKP) and 26 normal controls from a dry eye clinic at Seoul St. Mary’s Hospital between December 2014 and June 2017. This study was approved by the Institutional Review Board at Seoul St. Mary’s Hospital and conducted according to the ethical standards stated in the Declaration of Helsinki.

All participants underwent a comprehensive ophthalmologic examination, including measurement of visual acuity, slit-lamp biomicroscopy, Goldmann applanation tonometry, gonioscopic examinations, central corneal thickness using ultrasound pachymetry (Tomey Corporation, Nagoya, Japan), measurement of axial length, dilated stereoscopic examination of the optic nerve head, stereoscopic optic disc photography, red-free RNFL photography (VX-10; Kowa Optimed, Tokyo, Japan), Spectralis SD-OCT scans for measurement of RNFL, GCL, IPL and GCIPL thicknesses in the macular area, Cirrus (Carl Zeiss Meditec) SD-OCT for measurement of the thickness of the GCIPL in the macular area, and achromatic automated perimetry using the 24–2 Swedish Interactive Threshold Algorithm standard program (Humphrey Visual Field Analyzer; Carl Zeiss Meditec).

Participants had to meet the following inclusion criteria: best-corrected visual acuity ≥ 20/30, axial length within 30 mm and presence of a normal anterior chamber and an open angle. Eyes with unreliable VFs (defined as false negative > 15%, false positive > 15%, and fixation losses > 20%) were excluded. Patients with intraocular or neurologic disease that could cause VF loss or with any retinal disease, such as diabetic macular edema, epiretinal membrane, or age-related macular degeneration that could affect macular thickness and induce segmentation error were also excluded.

For a diagnosis of glaucoma, patients had to meet the following criteria: glaucomatous optic disc appearance that showed focal or diffuse rim thinning (e.g., notching or acquired pitting of the optic nerve) with visible RNFL defects on red-free photography and glaucomatous VF loss. The preperimetric glaucomatous eyes were defined as having a glaucomatous optic disc appearance in the presence of a normal VF, early perimetric glaucomatous eyes were defined as those with a mean deviation (MD) better than -6 dB, and moderate to advanced glaucomatous eyes were defined as those with MD worse than -6 dB, as confirmed by at least 2 reliable VF examinations. The stereophotograph assessment was confirmed and agreed by two glaucoma specialists (EKK, CKP).

### Optical coherence tomography

#### Spectralis SD-OCT

**Fig 1:**
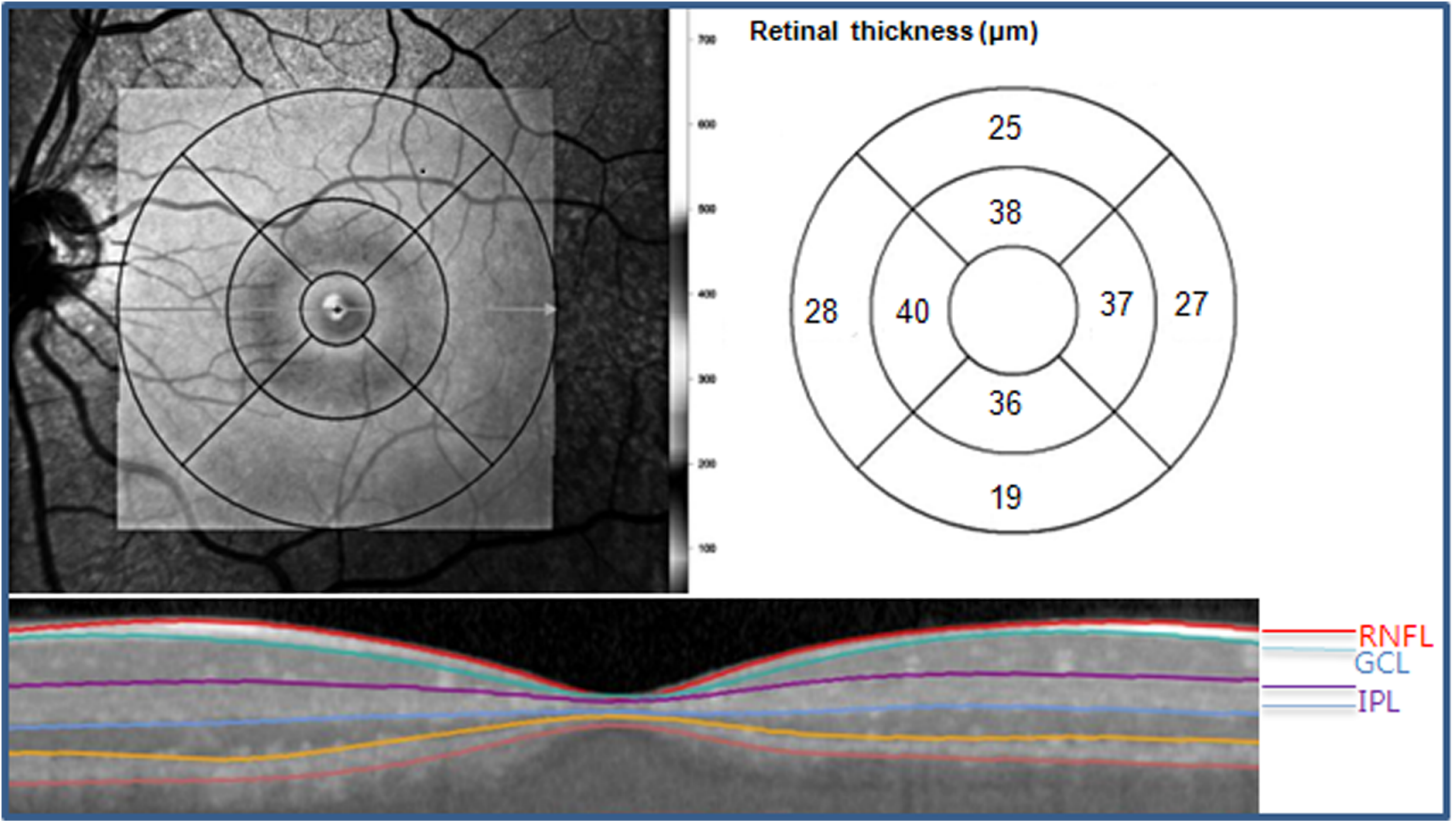
Divisions of the segmented retinal layer using Spectralis SD-OCT. Retinal thickness analysis protocol showing mean thicknesses for each of eight subfields: scan area of 6 × 6 mm, divided into three concentric circles with 1 mm, 3 mm, and 6 mm diameter, respectively. Here, we used values from the 3 and 6 mm circles of the grid excluding a central area (1 mm radius) that corresponded to the foveola. RNFL = retinal nerve fiber layer; GCL = ganglion cell layer; IPL = inner plexiform layer; SD-OCT = spectral-domain optical coherence tomography.

**Fig 2:**
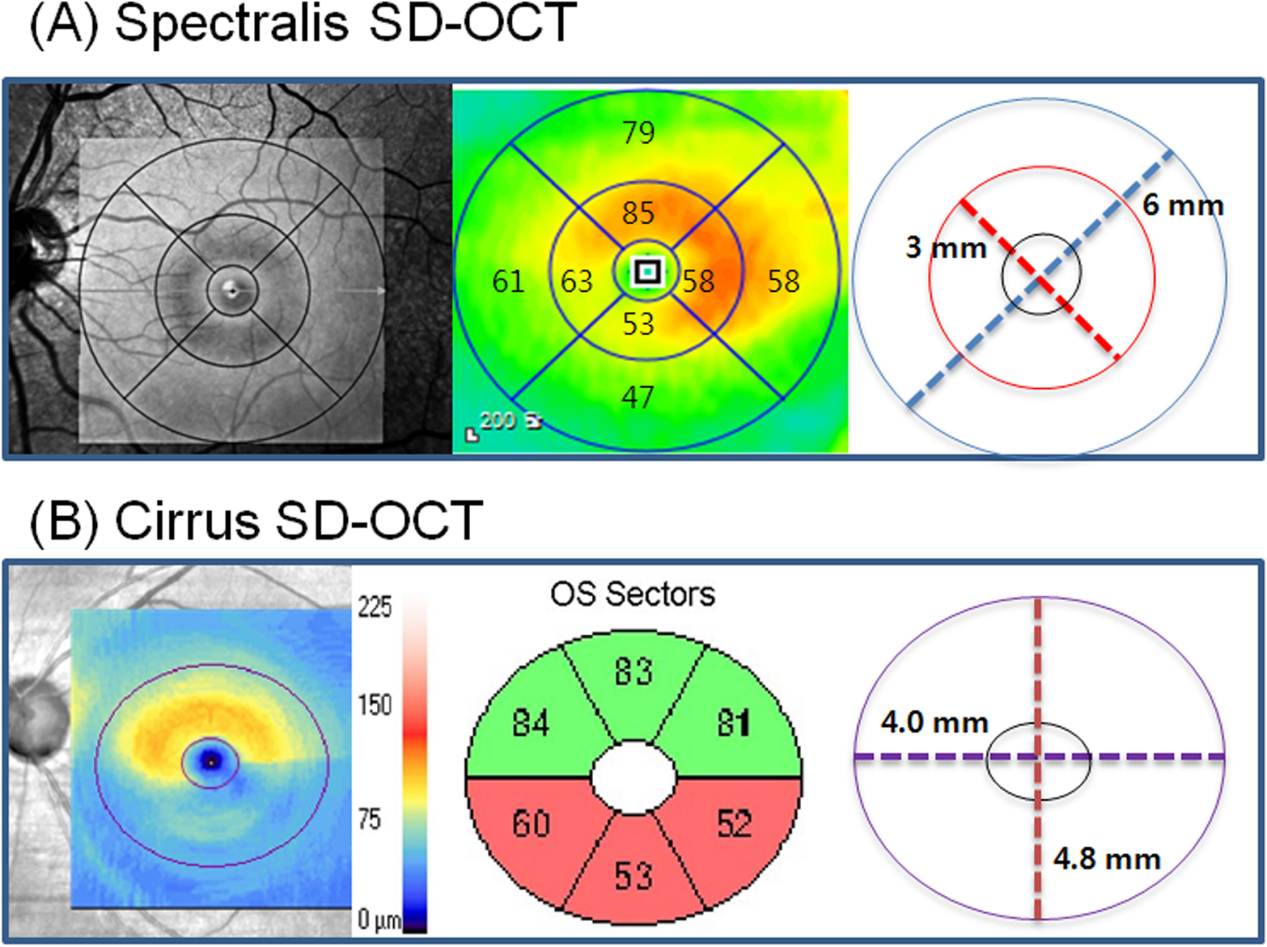
Printout taken from Spectralis and Cirrus SD-OCT. (A) Macular area used in Spectralis OCT to calculate average GCIPL thickness from the 3 and 6 mm circles of the grid. (B) Macular area used in Cirrus SD-OCT to calculate average GCIPL thickness corresponding to an elliptical annulus with a 2.0 mm vertical and 2.4 mm horizontal radius, excluding a central elliptical area (0.5 mm vertical and 0.6 mm horizontal radius) that corresponded to the foveola. GCIPL = ganglion cell-inner plexiform layer; SD-OCT, spectral-domain optical coherence tomography.

All patients were subjected to the “fast macular cube” protocols of the Spectralis SD-OCT. Spectralis SD-OCT incorporates real-time eye-tracking software (TruTrack; Heidelberg Engineering) has the advantage of improving image quality and segmentation accuracy to obtain retinal scans comprising 25 single horizontal axial scans in the macular area. The automated segmentation software for the Spectralis SD-OCT identified 4 different retinal boundaries: the inner limiting membrane, and the boundaries between the RNFL and the GCL, the GCL and the IPL, the IPL and the inner nuclear layer (INL). The retinal thickness map consists of three concentric rings with diameters of 1, 3, and 6 mm. The two outer rings are divided into quadrants by two intersecting lines. At the time of data acquisition, the intermediate and outer rings of diameters 3 and 6 mm, respectively, were considered for the analyses excluding a central area (1 mm radius) that corresponded to the foveola. The intermediate ring is divided into 4 zones, designated as the inner superior, inner nasal, inner inferior, and inner temporal, while the outer ring is divided into the outer superior, outer nasal, outer inferior, and outer temporal zones [14]. The average thickness of each layer was obtained by averaging four inner subfields and four outer subfields (Fig 1). For Bland-Altman analysis to validate the segmentation software of Spectralis SD-OCT, we calculated the GCIPL thickness using the GCL and IPL thicknesses. The GCIPL thickness was calculated as the sum of GCL and IPL thickness, and the average GCIPL thickness of 8 subfields was used in the analysis (Fig 2). All OCT scans were performed by the same experienced operator. The quality of the scans was assessed before analysis and the absence of movement artifact and well centered scans.

#### Cirrus SD-OCT

The three-dimensional macular cube scan was performed using the Cirrus SD-OCT algorithm for ganglion cell analysis for Bland-Altman analysis. The segmentation software of the Cirrus SD-OCT identified 3 different retinal boundaries: the inner limiting membrane, the outer boundary of the RNFL, and the outer boundary of the IPL. The distance from the internal limiting membrane to the outer boundary of the RNFL was defined as the RNFL thickness and the outer boundary of the RNFL to the outer boundary of the IPL was defined as the GCIPL thickness that is a combination of GCL and the IPL. The 6 sectoral (superotemporal, superior, superonasal, inferonasal, inferior, inferotemporal) thicknesses of the GCIPL were obtained in an elliptical annulus (dimensions, vertical inner and outer radius of 0.5 mm and 2.0 mm, horizontal inner and outer radius of 0.6 mm and 2.4 mm, respectively). Details of the manner in which GCIPL thickness is measured have been reported previous study [15]. The average GCIPL thickness of 6 sectors was used in the analysis (Fig 2).

#### Visual field examination

**Fig 3:**
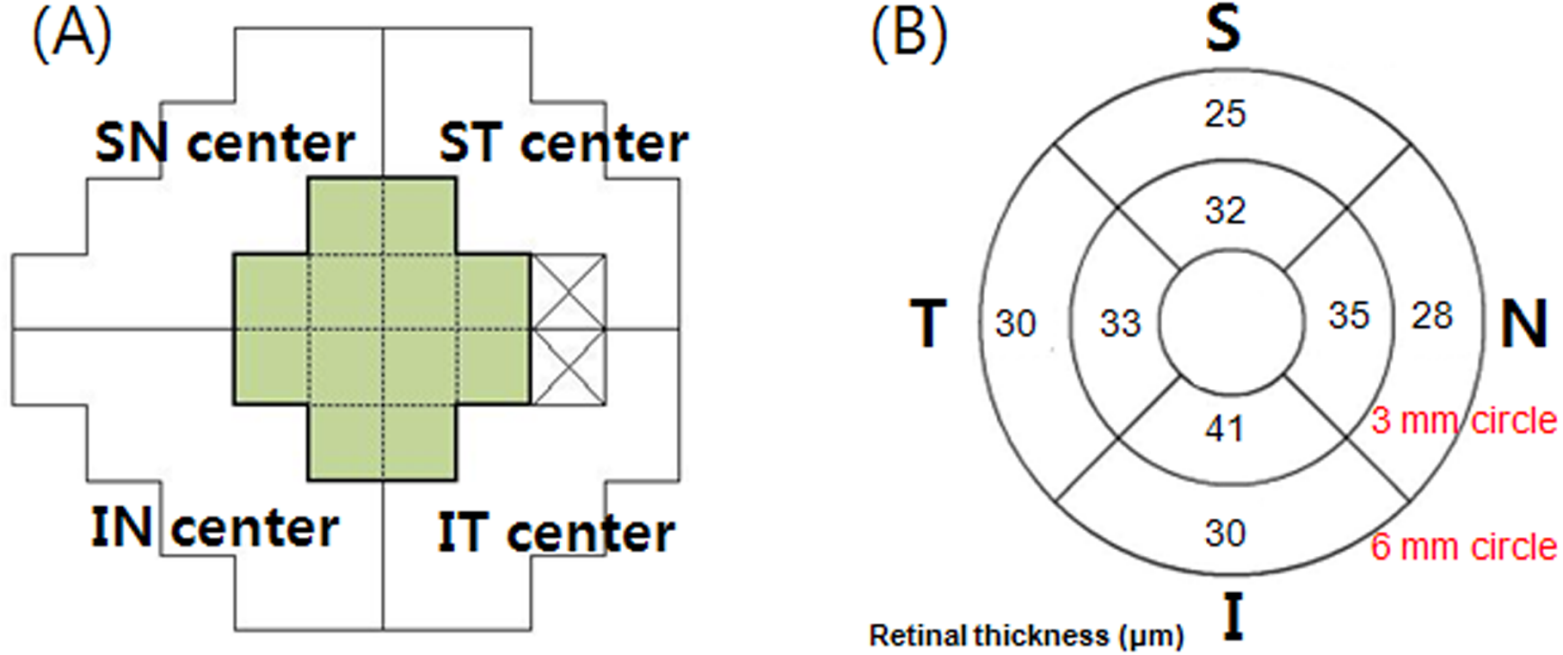
The VF of the Humphrey field analyzer Swedish interactive threshold algorithm 24–2 paradigm, segmentedretinal layer thickness measurements in macular area by Spectralis SD-OCT of a right eye. (A) The VF of the Humphrey field analyzer Swedish interactive threshold algorithm 24–2 paradigm. The corresponding central cluster MS was defined as the average of 12 central data points. (B) segmented retinal layer thickness measured by Spectralis SD-OCT (scan area of 6 × 6 mm, macular area) of a right eye. S = superior; ST = superotemporal; SN = superonasal; I = inferior; IT = inferotemporal; IN = inferonasal.

All patients underwent VF testing using the Swedish Interactive Threshold Algorithm Standard 24–2 strategy on the same day as the SD-OCT imaging. Structure-function relationships were analyzed by comparing the corresponding mean sensitivity (MS) values measured by 24–2 standard automated perimetry (SAP) and the OCT parameter assessed by Spectralis SD-OCT. A glaucomatous VF defect was defined as a cluster of 3 or more points with a probability of < 5% on the pattern deviation map in at least 1 hemifield, including at least 1 point with a probability of < 1%; a result outside normal limits in the glaucoma hemifield test; or a pattern standard deviation with a probability of < 5%. Two test points located in the blind spot were excluded from the analysis. The corresponding area for VF, which was assumed to correspond topographically to the retina within 6.0 mm of the fovea, was defined as the average of 12 central cluster points according to the structure–function correspondence map suggested by Garway-Heath et al (Fig 3) [16,17].

### Statistical analysis

Age, intraocular pressure, central corneal thickness, spherical equivalent, axial length, mean deviation, pattern standard deviation, and average RNFL, GCL, and IPL thicknesses were compared among pre-perimetric glaucoma, early glaucoma, and moderate to advanced glaucoma eyes using Kruskal-Wallis analysis. Correlations between the thicknesses of the RNFL, GCL, IPL, and GCIPL and the corresponding MS values of VF were evaluated using regression analyses and locally-weighted scatterplot smoothing (lowess) fit. Lowess fit is non-parametric regression to minimize the noise while avoiding assumptions about the relationship among variables [18]. Inter-instrument reproducibility of retinal thickness by Cirrus OCT and Spectralis OCT was evaluated by intraclass correlation coefficients (ICCs) and Bland-Altman plots with limits of agreement (LoA) for qualification of the performance of the automated segmentation ability. The statistical analysis was performed using Statistical Package for the Social Sciences software (SPSS Inc., Chicago, IL, USA). A P-value < .05 was considered to be statistically significant.

## Results

Participants in this study were 46 male and 65 female Asian. In total, 26 normal controls, 26 preperimetric glaucomatous eyes, 26 early glaucomatous eyes, and 33 moderate to advanced glaucomatous eyes were enrolled in this cross-sectional study. Preperimetric glaucoma had glaucomatous optic neuropathy without VF abnormalities and early glaucoma eyes and moderate to advanced glaucoma had glaucomatous VF abnormalities. The demographics of subjects are summarized in Table 1. We found no intergroup difference in terms of age, intraocular pressure, central corneal thickness, spherical equivalent, or axial length. The VF parameters for the 4 groups are also compared in Table 1. The mean deviation was -0.56 ± 0.81 dB in preperimetric glaucomatous eyes, -3.95 ± 0.77 dB in early glaucomatous eyes, and -9.66 ± 3.61 dB in moderate to advanced glaucomatous eyes. There were significant differences in MD and PSD among the 4 groups (P < .001).

**Table 1:** Demographics and ocular characteristics of participants with glaucoma and normal controls.

	Normal controls	Pre-perimetric glaucoma	Early glaucoma	Moderate to advanced glaucoma	*P* value
Subject eyes (n)	26	26	26	33	
Mean age (y)	54.12 ± 12.75	52.08 ± 11.77	55.65 ± 13.36	51.33 ± 13.78	.527^*^
Gender ratio, male:female	10: 16	10:16	10:16	16:17	.682^†^
Intraocular pressure (mmHg)					
Baseline	16.81 ± 5.68	15.92 ± 3.53	16.62 ± 5.01	15.82 ± 3.68	.787^*^
Mean during follow-up	16.42 ± 4.98	13.42 ± 3.20	13.27 ± 2.83	13.40 ± 2.97	.993^*^
Central corneal thickness (μm)	528.32 ± 37.16	537.92 ± 36.50	537.48 ± 38.51	527.35 ± 28.20	.410^*^
Spherical equivalent (D)	-0.87 ± 2.40	-2.22 ± 3.40	-1.41 ± 3.24	-3.03 ± 3.71	.238^*^
Axial length (mm)	23.78 ± 1.44	24.88 ± 1.56	24.05 ± 1.55	24.81 ± 1.77	.201^*^
Mean MD of 24–2 VF (dB)	-0.3 ± 0.47	-0.56 ± 0.81	-3.95 ± 0.77	-9.66 ± 3.61	< .001^*^
Mean PSD of 24–2 VF (dB)	1.42 ± 0.21	1.62 ± 0.33	5.73 ± 2.04	10.92 ± 4.17	< .001^*^

Data are presented as the mean and standard deviation.

^*^Comparison between the three groups by Kruskal-Wallis one-way analysis of variance.

^†^Comparison between the three groups by Chi-square test.

MD = mean deviation; PSD = pattern standard deviation; VF = visual field.

**Fig 4:**
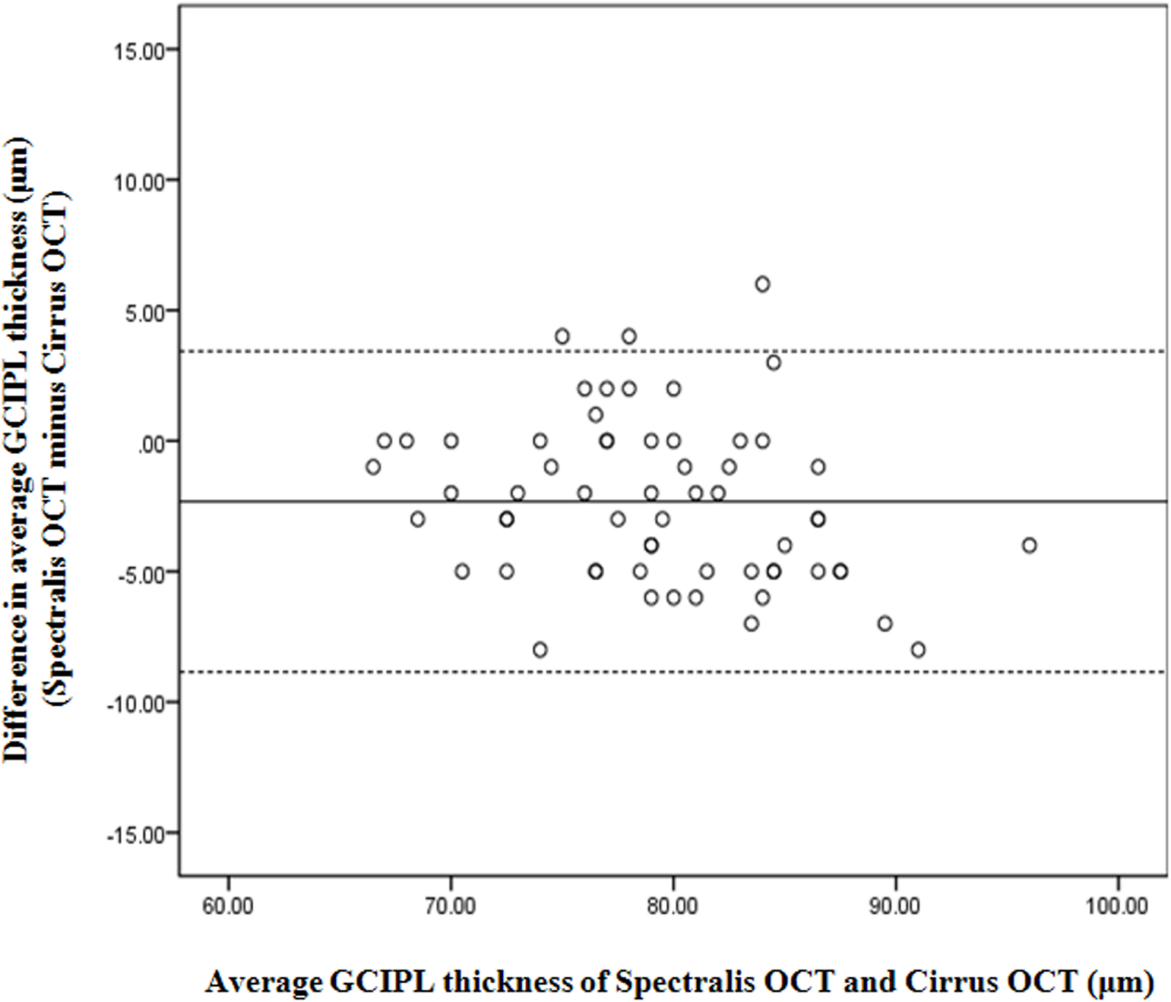
Bland-Altman analysis showing distribution of differences in GCIPL thickness between Spectralis and Cirrus SD-OCT. The y-axis represents Spectralis value minus Cirrus value (μm) and x-axis represents average of GCIPL thickness values of 2 SD-OCT (μm) for each eye. Mean differences between the two devices are 2.29 μm in the macular area. GCIPL = ganglion cell-inner plexiform layer; SD-OCT, spectral-domain optical coherence tomography.

To validate the segmentation software, we compared the average GCIPL thickness of the Spectralis SD-OCT with those of the Cirrus SD-OCT. There was excellent agreement between Spectralis and Cirrus SD-OCT for segmentation ability (exact agreement 96.2%; P < .001). A Bland-Altman scatter plot comparing the Spectralis and Cirrus SD-OCT (Fig 4) also showed excellent agreement between the 2 instruments. The differences of GCIPL thickness had a mean of 2.69 µm in the macular region.

**Fig 5:**
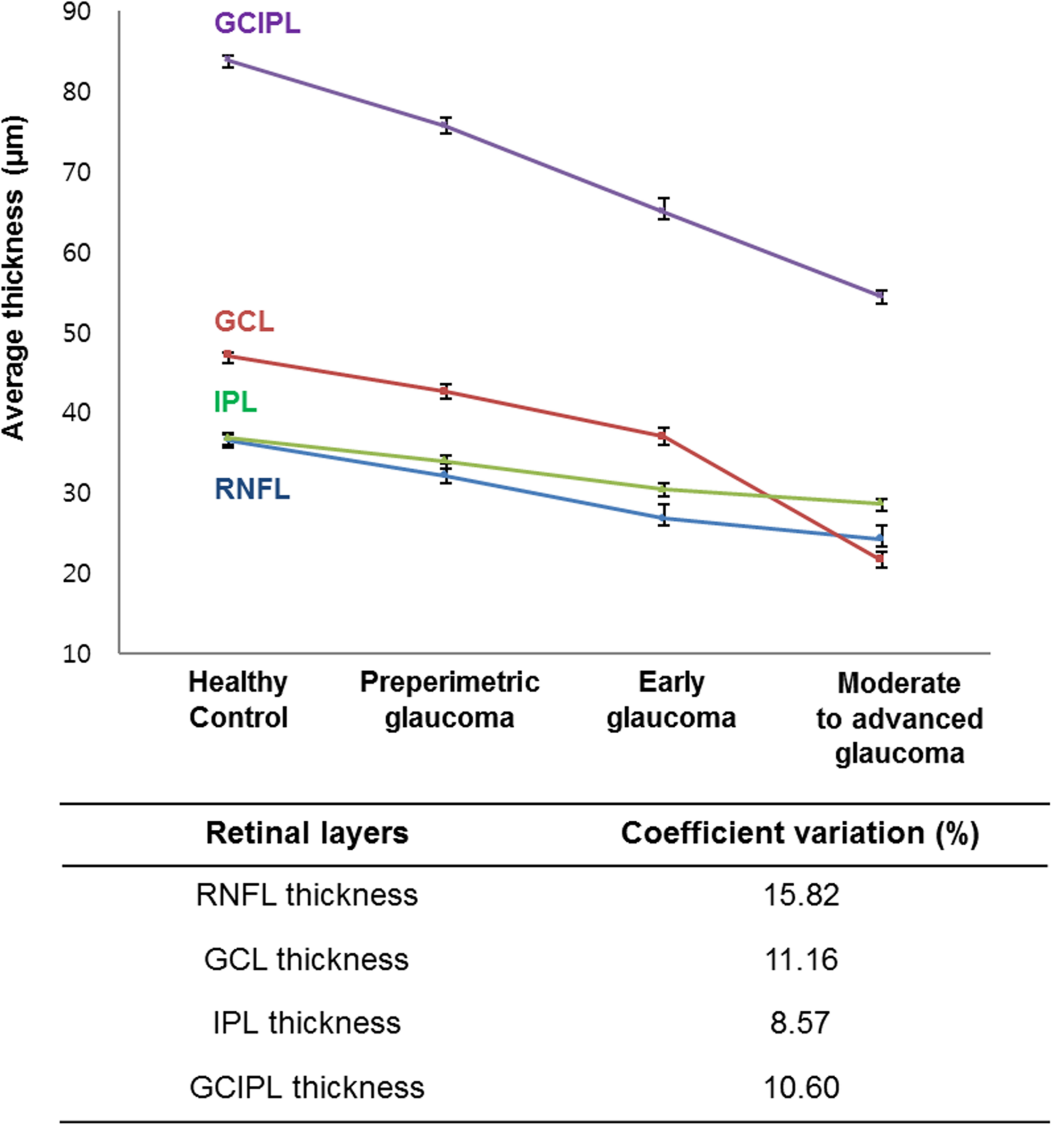
Average RNFL, GCL, IPL and GCIPL thicknesses in the macular region and coefficient variations of segmented layer measurements. RNFL = retinal nerve fiber layer; GCL = ganglion cell layer; IPL = inner plexiform layer; GCIPL = ganglion cell-inner plexiform layer.

**Fig 6:**
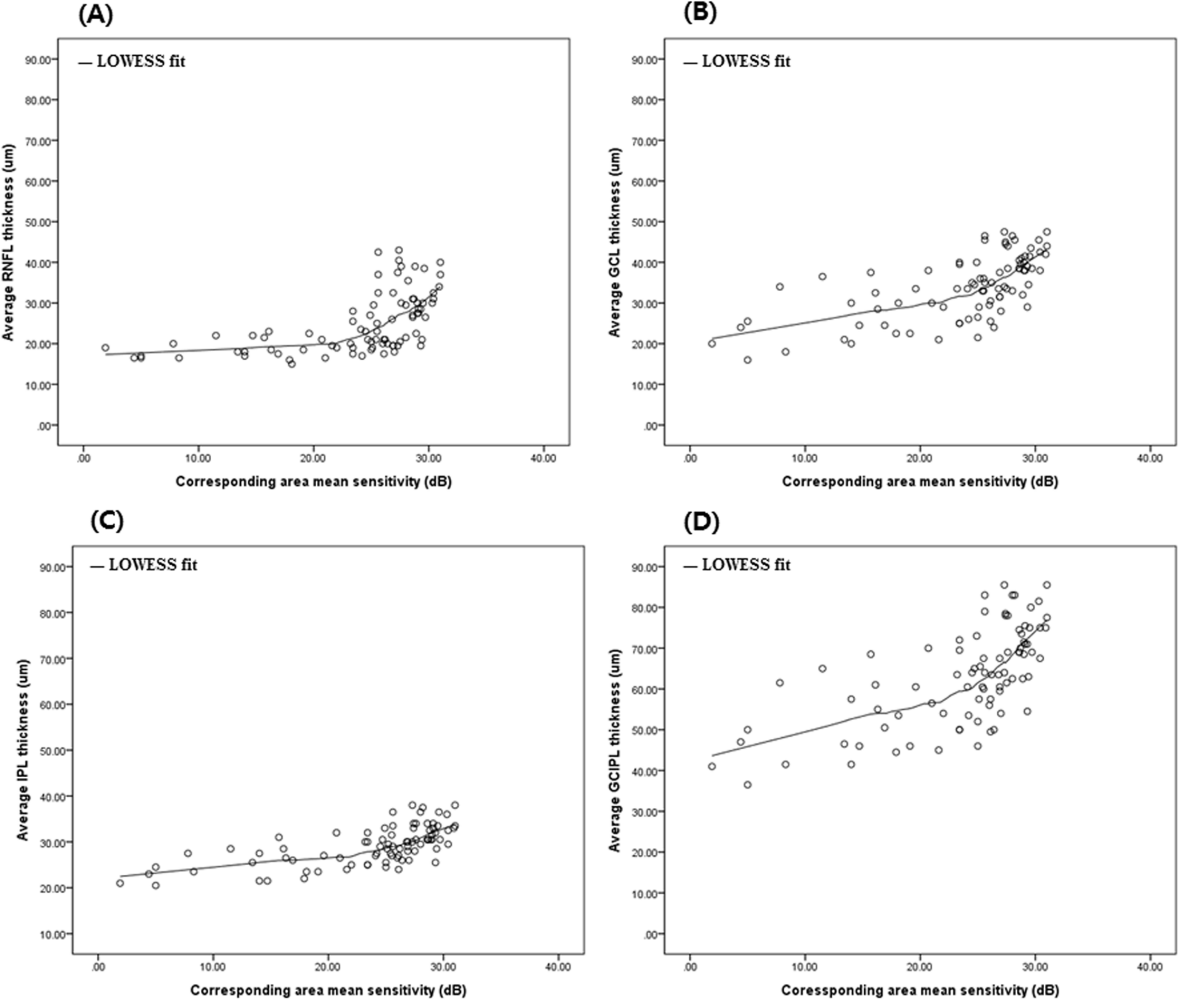
Scatter plots showing the correlation between macular inner retinal thickness and corresponding mean sensitivity value in all glaucoma groups. Note the scaling for the y-axis, where thickness of inner retina is expressed in micrometers, and also that the x-axis indicates VF mean sensitivity (dB) of the corresponding area. (A) Average RNFL thickness versus central corresponding area MS (dB). (B) Average GCL thickness versus central corresponding area MS (dB). (C) Average IPL thickness versus central corresponding area MS (dB). (D) Average GCIPL thickness versus central corresponding area MS (dB). RNFL = retinal nerve fiber layer; GCL = ganglion cell layer; IPL = inner plexiform layer; GCIPL = ganglion cell-inner plexiform layer; MS = mean sensitivity; VF = visual field.

**Fig 7:**
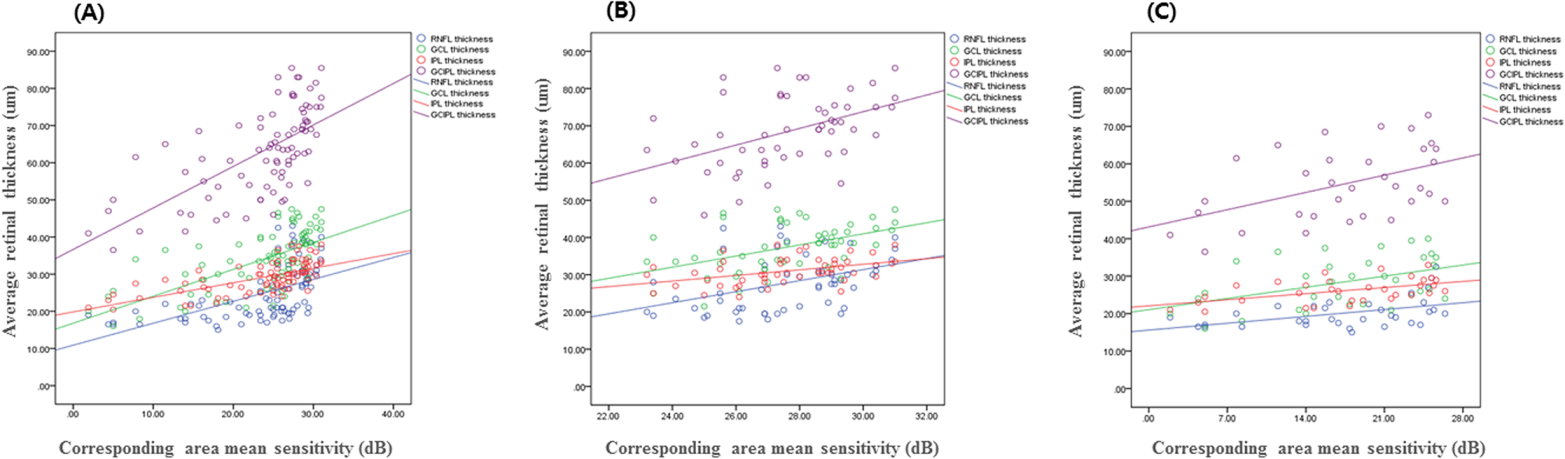
Scatter plots showing the correlation between macular inner retinal thickness and corresponding mean sensitivity value in glaucoma groups. Note the scaling for the y-axis, where thickness of inner retina is expressed in micrometers, and also that the x-axis indicates VF mean sensitivity (dB) of the corresponding area. (A) Average RNFL, GCL, IPL and GCIPL thickness versus central corresponding area MS (dB) in all glaucoma groups (R^2^ = 0.306, P< .001 for RNFL thickness; R^2^ = 0.404, P< .001 for GCL thickness; R^2^ = 0.420, P< .001 for IPL thickness; R^2^ = 0.417, P< .001 for GCIPL thickness). (B) Average RNFL, GCL, IPL and GCIPL thickness versus central corresponding area MS (dB) in pre-perimetric and early glaucoma (R^2^ = 0.151, P = .003 for RNFL thickness; R^2^ = 0.210, P = .001 for GCL thickness; R^2^ = 0.192, P< .001 for IPL thickness; R^2^ = 0.205, P< .001 for GCIPL thickness). (C) Average RNFL, GCL, IPL and GCIPL thickness versus central corresponding area MS (dB) in moderate to advanced glaucoma (R^2^ = 0.216, P = .006 for RNFL thickness; R^2^ = 0.204, P = .008 for GCL thickness; R^2^ = 0.266, P = .002 for IPL thickness; R^2^ = 0.234, P = .004 for GCIPL thickness). RNFL = retinal nerve fiber layer; GCL = ganglion cell layer; IPL = inner plexiform layer; GCIPL = ganglion cell-inner plexiform layer; MS = mean sensitivity; VF = visual field.

The RNFL, GCL, IPL, and GCIPL thicknesses in the 4 groups are provided in Table 2. There were statistically significant differences in thickness of the RNFL, GCL, IPL and GCIPL among the 4 groups. Table 3 shows the structure-function correlations of the inner retinal thickness in patients with glaucoma. There was a significant correlation between the corresponding MS value for VF and thickness of the RNFL, GCL, IPL and GCIPL (R = 0.553, 0.636, 0.648, and 0.646 respectively, P < .001). Fig 5 displays the schematic changes of 3 inner retinal layers at different stages of glaucoma, and coefficient variations of 3 these layers. Fig 6 shows the locally-weighted scatterplot smoothing (LOWESS) plot suggests a curvilinear relationship between the corresponding MS value for the VF and the average thickness of each layer. In Fig 7, details of structure-function relationship, and the analysis for each stage is shown. The R squared values of 4 inner retinal thicknesses were statistically significant in all glaucoma groups (R^2^ = 0.306, P< .001 for RNFL thickness; R^2^ = 0.404, P< .001 for GCL thickness; R^2^ = 0.420, P< .001 for IPL thickness; R^2^ = 0.417, P< .001 for GCIPL thickness).

**Table 2:** Average RNFL, GCL, IPL and GCIPL thicknesses in the macular area of normal controls, pre-perimetric glaucoma, early glaucoma, and moderate to advanced glaucoma.

	Healthy Control (A)	Pre-perimetric glaucoma (B)	Early glaucoma (C)	Moderate to advanced glaucoma (D)	*P* value^*^
Average RNFL thickness	36.63±2.33	32.16±5.13	26.84±7.87	24.31±3.64	< .001
Post hoc comparison (*P* value)		A vs B (< .001)	A vs C (< .001)	A vs D (< .001)	
			B vs C (< .001)	B vs D (< .001)	
				C vs D (0.032)	
Average GCL thickness	47.13±1.89	42.63±4.20	37.02±5.18	21.67±5.29	< .001
Post hoc comparison (*P* value)		A vs B (< .001)	A vs C (< .001)	A vs D (< .001)	
			B vs C (< .001)	B vs D (< .001)	
				C vs D (0.02)	
Average IPL thickness	36.91±2.12	34.01±2.77	30.49±3.14	28.70±3.12	< .001
Post hoc comparison (*P* value)		A vs B (< .001)	A vs C (< .001)	A vs D (< .001)	
			B vs C (< .001)	B vs D (< .001)	
				C vs D (0.01)	
Average GCIPL thickness	84.03±3.72	75.75±7.45	65.09±8.82	54.57±9.64	< .001
Post hoc comparison (*P* value)		A vs B (< .001)	A vs C (< .001)	A vs D (< .001)	
			B vs C (< .001)	B vs D (< .001)	
				C vs D (0.01)	

*Comparison between the three groups by Kruskal-Wallis one-way analysis of variance.

RNFL = retinal nerve fiber layer; GCL = ganglion cell layer; IPL = inner plexiform layer; GCIPL = ganglion cell-inner plexiform layer.

**Table 3:** Comparisons of relationships between inner retinal thicknesses in the macular area and corresponding visual field sensitivities*.

	Correlation coefficient	p-value
Central cluster MS, dB		
Average RNFL thickness	.553	< .001
Average GCL thickness	.636	< .001
Average IPL thickness	.648	< .001
Average GCIPL thickness	.646	< .001

^*^Corresponding mean sensitivity value (dB) was measured by 24–2 SAP.

RNFL = retinal nerve fiber layer; GCL = ganglion cell layer; IPL = inner plexiform layer; GCIPL = ganglion cell-inner plexiform layer.

## Discussion

It is meaningful to observe changes of RGCs, which are the key component of the pathophysiology of glaucoma. In this study, we examined how RNFL, GCL, and IPL changed in glaucoma and demonstrated that the IPL, in which even though the neuronal fibers of three different cells are mixed, decreases in thickness as glaucoma progresses. This suggests that many dendritic changes occur in the RGC in patients with glaucoma. Furthermore, we found a correlation between loss of IPL thickness and loss of visual function. Consistent with our clinical findings, Della Santina et al reported that injury to RGC axons triggers rapid pathologic changes in RGC dendrites, including branch retraction, reduced complexity, and synaptic loss in experimental glaucoma model [19–21].

In pre-perimetric and early glaucoma, the structure-function relationship of the GCL, IPL, and GCIPL thickness was stronger than the RNFL thickness (Fig 7). In a similar vein, Mayama et al reported that macular GCIPL was more useful than macular RNFL or peripapillary RNFL for diagnosing early-stage glaucoma [22]. In our study, we demonstrated that glaucomatous changes processed not only in the GCL, representing the RGC soma, but also in the IPL, representing RGC dendrites in pre-perimetric and early glaucoma. Frankfort et al reported that glaucoma-induced damage to the RGC causes axonal and somatic changes as well as dendrite, and morphologic changes in RGC dendrites were detected slightly prior to axonal thinning or soma shrinkage, suggesting that dendritic abnormalities precede degeneration of other ganglion cell compartments [23]. Similary, Rana et al reported that dendrites are the first structures to become altered in the early stages of glaucoma and are therefore more vulnerable to glaucomatous damage [24]. These studies indicate that dendritic changes might be used as a valuable biomarker to measure RCG dysfunction and neuro-degeneration in early stage of glaucoma.

A number of studies have used OCT to show that there are significant strong correlations between SAP-determined VF sensitivity and OCT-determined macular GCIPL thickness [25–29]. GCIPL thickness may be valuable for structural evaluation of the macular region and for understanding the structure-function relationship in glaucoma. In our study, we analyzed GCIPL as GCL and IPL, and demonstrated that IPL and GCIPL thicknesses showed greatest structure-function relationship of the 4 inner retinal layer thicknesses throughout the course of glaucoma (Table 3). Raza et al have published virtually the only clinical study using IPL thickness and reported a significant reduction of macular IPL thickness in patients with severe glaucoma [30]. As far as we know, our study is the first to clinically analyze the structure-function relationship in IPL, and these segmented IPL parameters could be useful for diagnosis of glaucoma and progression monitoring of disease.

In addition, we investigated for a correlation between the 4 inner retinal layer thicknesses and the corresponding VF sensitivity using a scatter plot. In Fig 6, lowess plot suggested a curvilinear relationship between VF sensitivity and RNFL, GCL, IPL, and GCIPL thickness as previous reports [28]. In this study, we used logarithmic decibel scale for functional evaluation and the study population included patients with mild VF defects at a high rate, therefore we performed lowess fit to show the structure-function relationship. We also analyzed R-square value using multivariate linear regression analysis for each glaucoma stage in Fig 7. We observed that IPL and GCIPL thicknesses showed stronger structure-function relationships compared with RNFL and GCL thicknesses, and association values for IPL thickness and GCIPL thickness were comparable. The GCIPL thickness included both GCL thickness which showed strongest structure-function correlation in early glaucoma, and IPL thickness which showed strongest structure-function correlation in moderate to advanced glaucoma. For this reason, the correlation of GCIPL thickness showed almost identical association values in all glaucoma groups. Regarding coefficient variations of segmented layers, IPL thickness displayed a fairly narrow confidence interval over the progression of glaucoma. Thus we suggest that considering IPL thickness along with GCIPL thickness could be clinically useful for evaluating all stages of glaucoma.

There are several limitations to this study. First, the automated segmentation software used in our study presented some problems. For example, artifacts, such as the light reflex, vitreous separation, or epiretinal membranes, can cause local retinal thickening. Also, a thin RNFL in and around the horizontal Raphe, as well as the inner retinal layers in the fovea, can cause problems when using automated algorithms. To minimize the segmentation error, we performed repeated segmentations and checked the segmented layer manually. Second, using the central 10–2 VF may be more appropriate for evaluation of the relationship between VF sensitivity and macular RNFL, GCL, and IPL. It is well known that the 10–2 SAP test provides more valuable information than the 24–2 SAP test in the patient with a parafoveal field defect [31–33]. Instead of the 10–2 SAP, we selected 12 central cluster VF points using the 24–2 SAP, which is the most commonly used VF examination. In our study, using the 24–2 SAP test, we demonstrated that the relationship between the central corresponding MS and IPL thickness was significantly stronger than that for RNFL or GCL thickness. Third, this cross-sectional study did not include longitudinal structural and functional data. Therefore, a longitudinal study should be performed to correlate inner retinal changes in the macular region with functional status in glaucomatous optic neuropathy. Lastly, there might be an abundance of retinal capillaries in IPL besides RGC dendrites, axons of bipolar cells, and processes of amacrine cells. Campbell et. al reported that retinal capillary plexuses are vertically and horizontally distributed from RNFL to outer plexiform layer (OPL) forming superficial and deep network [34]. Other contributing factors, a retinal vasculature, vascular tone, and perfusion differences, to affect IPL thickness change should be considered.

In conclusion, segmental analysis of the inner retinal layer including the IPL may provide further valuable information for understanding the RGC changes that occur in glaucoma. We have demonstrated that the IPL, even though the neuronal fibers of three different cells are mixed in, shows stronger structure-function relationship compared with the RNFL and the GCL during the course of glaucoma. Further, our findings indicate that the IPL thickness was significantly associated with the degree of glaucoma and can be used as a potential biomarker to evaluate glaucoma. Finally we carefully suggest that clinicians should consider IPL thickness in the macular area when diagnosing glaucoma and monitoring for progression of the disease along with other inner retinal layer thicknesses. This knowledge will likely be useful for the development of strategies to maintain or promote the stability of dendrites and to preserve visual function in glaucoma.

## Supporting information

S1 Table Demographics and ocular characteristics of participants with glaucoma and normal controls. (DOCX)

S2 Table Average RNFL, GCL, IPL and GCIPL thicknesses in the macular area of normal controls, pre-perimetric glaucoma, early glaucoma, and moderate to advanced glaucoma. (DOCX)

S3 Table Comparisons of relationships between inner retinal thicknesses in the macular area and corresponding visual field sensitivities. (DOCX)
