# Supplementary material for: Segmented inner plexiform layer thickness as a potential biomarker to evaluate open-angle glaucoma: Dendritic degeneration of retinal ganglion cell
Source: PLoS One. 2017 Aug 3;12(8):e0182404. doi: 10.1371/journal.pone.0182404 (PMC5542626; doi:10.1371/journal.pone.0182404)
Supplement: S1 Table — (DOCX) [file pone.0182404.s001.docx]

**Table 1.**

|  | **Healthy control** | **Pre-perimetric glaucoma** | **Early glaucoma** | **Moderate to advanced glaucoma** | *P* value |
| --- | --- | --- | --- | --- | --- |
|  |  |  |  |  |  |
| Subject eyes (n) | 26 | 26 | 26 | 33 |  |
| Mean age (y) | 54.12 ± 12.75 | 52.08 ± 11.77 | 55.65 ± 13.36 | 51.33 ± 13.78 | .527^*^ |
| Gender ratio, male:female | 10 :16 | 10:16 | 10:16 | 16:17 | .682^†^ |
| Intraocular pressure (mmHg) |  |  |  |  |  |
| Baseline | 16.81 ± 5.68 | 15.92 ± 3.53 | 16.62 ± 5.01 | 15.82 ± 3.68 | .787^*^ |
| Mean during follow-up | 16.42 ± 4.98 | 13.42 ± 3.20 | 13.27 ± 2.83 | 13.40 ± 2.97 | .993^*^ |
| Central corneal thickness (μm) | 528.32 ± 37.16 | 537.92 ± 36.50 | 537.48 ± 38.51 | 527.35 ± 28.20 | .410^*^ |
| Spherical equivalent (D) | -0.87 ± 2.40 | -2.22 ± 3.40 | -1.41 ± 3.24 | -3.03 ± 3.71 | .238^*^ |
| Axial length (mm) | 23.78 ± 1.44 | 24.88 ± 1.56 | 24.05 ± 1.55 | 24.81 ± 1.77 | .201^*^ |
| Mean MD of 24-2 VF (dB) | -0.3 ± 0.47 | -0.56 ± 0.81 | -3.95 ± 0.77 | -9.66 ± 3.61 | < .001^*^ |
| Mean PSD of 24-2 VF (dB) | 1.42 ± 0.21 | 1.62 ± 0.33 | 5.73 ± 2.04 | 10.92 ± 4.17 | < .001^*^ |

**Data are presented as the mean and standard deviation.**

**^*^Comparison between the three groups by Kruskal-Wallis one-way analysis of variance.**

**^†^Comparison between the three groups by Chi-square test.**

**MD = mean deviation; PSD = pattern standard deviation; VF = visual field.**
