# Supplementary material for: Segmented inner plexiform layer thickness as a potential biomarker to evaluate open-angle glaucoma: Dendritic degeneration of retinal ganglion cell
Source: PLoS One. 2017 Aug 3;12(8):e0182404. doi: 10.1371/journal.pone.0182404 (PMC5542626; doi:10.1371/journal.pone.0182404)
Supplement: S2 Table — (DOCX) [file pone.0182404.s002.docx]

**Table 2.**

|  | **Healthy**  **Control (A)** | **Pre-perimetric glaucoma (B)** | **Early**  **glaucoma (C)** | **Moderate to advanced glaucoma (D)** | *P* value^*^ |
| --- | --- | --- | --- | --- | --- |
|  |  |  |  |  |  |
| Average RNFL thickness | 36.63±2.33 | 32.16±5.13 | 26.84±7.87 | 24.31±3.64 | <.001 |
| Post hoc comparison (*P* value) |  | A vs B (<.001) | A vs C (<.001) | A vs D (<.001) |  |
|  |  |  | B vs C (<.001) | B vs D (<.001) |  |
|  |  |  |  | C vs D (0.032) |  |
| Average GCL thickness | 47.13±1.89 | 42.63±4.20 | 37.02±5.18 | 21.67±5.29 | <.001 |
| Post hoc comparison (*P* value) |  | A vs B (<.001) | A vs C (<.001) | A vs D (<.001) |  |
|  |  |  | B vs C (<.001) | B vs D (<.001) |  |
|  |  |  |  | C vs D (0.02) |  |
| Average IPL thickness | 36.91±2.12 | 34.01±2.77 | 30.49±3.14 | 28.70±3.12 | <.001 |
| Post hoc comparison (*P* value) |  | A vs B (<.001) | A vs C (<.001) | A vs D (<.001) |  |
|  |  |  | B vs C (<.001) | B vs D (<.001) |  |
|  |  |  |  | C vs D (0.01) |  |
| Average GCIPL thickness | 84.03±3.72 | 75.75±7.45 | 65.09±8.82 | 54.57±9.64 | <.001 |
| Post hoc comparison (*P* value) |  | A vs B (<.001) | A vs C (<.001) | A vs D (<.001) |  |
|  |  |  | B vs C (<.001) | B vs D (<.001) |  |
|  |  |  |  | C vs D (0.01) |  |

***Comparison between the three groups by Kruskal-Wallis one-way analysis of variance.**

**RNFL = retinal nerve fiber layer; GCL = ganglion cell layer; IPL = inner plexiform layer; GCIPL = ganglion cell-inner plexiform layer.**
