# Supplementary material for: Segmented inner plexiform layer thickness as a potential biomarker to evaluate open-angle glaucoma: Dendritic degeneration of retinal ganglion cell
Source: PLoS One. 2017 Aug 3;12(8):e0182404. doi: 10.1371/journal.pone.0182404 (PMC5542626; doi:10.1371/journal.pone.0182404)
Supplement: S3 Table — (DOCX) [file pone.0182404.s003.docx]

**Table 3.**

|  | Correlation coefficient | p-value |
| --- | --- | --- |
| Central cluster MS, dB |  |  |
| Average RNFL thickness | .553 | <.001 |
| Average GCL thickness | .636 | <.001 |
| Average IPL thickness | .648 | <.001 |
| Average GCIPL thickness | .646 | <.001 |

**^*^Corresponding mean sensitivity value (dB) was measured by 24-2 SAP.**

**RNFL = retinal nerve fiber layer; GCL = ganglion cell layer; IPL = inner plexiform layer; GCIPL = ganglion cell-inner plexiform layer.**
